# Genome-wide analysis of long non-coding RNAs in adult tissues of the melon fly, *Zeugodacus cucurbitae* (Coquillett)

**DOI:** 10.1186/s12864-020-07014-x

**Published:** 2020-08-31

**Authors:** Wei-Jun Li, Yu-Jia Song, Hong-Liang Han, Hui-Qian Xu, Dong Wei, Guy Smagghe, Jin-Jun Wang

**Affiliations:** 1 Chongqing Key Laboratory of Entomology and Pest Control Engineering, College of Plant Protection, Southwest University, Chongqing, 400715 China; 2 International Joint Laboratory of China-Belgium on Sustainable Crop Pest Control, State Cultivation Base of Crop Stress Biology for Southern Mountainous Land, Academy of Agricultural Sciences, Southwest University, Chongqing, 400715 China; 3 Department of Plants and Crops, Ghent University, 9000 Ghent, Belgium

**Keywords:** Long non-coding RNA, *Zeugodacus cucurbitae*, RNA-seq, Transcriptome, Differential expressions

## Abstract

**Background:**

Long non-coding RNAs (lncRNAs) are involved in many fundamental biological processes, such as transcription regulation, protein degradation, and cell differentiation. Information on lncRNA in the melon fly, *Zeugodacus cucurbitae* (Coquillett) is currently limited.

**Results:**

We constructed 24 RNA-seq libraries from eight tissues (midgut, Malpighian tubules, fat body, ovary, and testis) of *Z. cucurbitae* adults. A total of 3124 lncRNA transcripts were identified. Among those, 1464 were lincRNAs, 1037 were intronic lncRNAs, 301 were anti-sense lncRNAs, and 322 were sense lncRNAs. The majority of lncRNAs contained two exons and one isoform. Differentially expressed lncRNAs were analyzed between tissues, and Malpighian tubules versus testis had the largest number. Some lncRNAs exhibited strong tissue specificity. Specifically expressed lncRNAs were identified and filtered in tissues of female and male *Z. cucurbitae* based on their expression levels. Four midgut-specific lncRNAs were validated by quantitative real-time polymerase chain reaction (RT-qPCR), and the data were consistent with RNA-seq data. Gene Ontology (GO) and Kyoto Encyclopedia of Genes and Genomes (KEGG) pathway analyses of targets of midgut-specific lncRNAs indicated an enrichment of the metabolic process.

**Conclusions:**

This was the first systematic identification of lncRNA in the melon fly. Expressions of lncRNAs in multiple adult tissues were evaluated by quantitative transcriptomic analysis. These qualitative and quantitative analyses of lncRNAs, especially the tissue-specific lncRNAs in *Z. cucurbitae*, provide useful data for further functional studies.

## Background

The high-throughput sequencing technology has greatly stimulated studies of insect genomes and transcriptomes [1]. Hundreds of insect genomes and transcriptomes are now accessible in the NCBI Short Read Archive (SRA) database. These provide valuable information for gene annotation [2]. As the member of the non-coding RNA families, long non-coding RNA (lncRNA) is defined as transcript longer than 200 nt (nucleotides) without protein-coding potential [3, 4]. Non-coding RNAs play essential roles in many biological processes, such as genomic imprinting, dosage compensation, and post-transcription regulation [5, 6]. However, most studies of insect transcriptome analysis have focused on protein-coding genes, and non-coding RNAs were less informative [7]. According to the genomic location [8], lncRNAs are classified into four subcategories: long intergenic non-coding RNA (lincRNA), sense lncRNA, anti-sense lncRNA, and intronic lncRNA [9]. In eukaryotes, lncRNAs are transcribed at several sites of the genome by RNA polymerase II and RNA polymerase III [10]. Similar to mRNAs, lncRNAs are modulated by post-transcriptional modifications, such as polyadenylation, splicing, and capping [11]. LncRNAs show poor conservation among different species and have relatively low expression level compared with mRNAs [12].

Systematic identification and analyses of lncRNAs have been investigated in various species, such as goat [13], mouse [14], zebrafish [15], tilapia [4], chicken [16], and fungus [17]. Many studies have provided data enabling lncRNA identification in insects. In *Drosophila melanogaster*, a total of 1875 candidate lncRNAs were identified from multiple transcriptome data sets [18]. Using RNA-seq technology, 8096 putative lncRNAs were identified in one susceptible and two insecticide-resistant strains of *Plutella xylostella* [19]. In addition, 2949 lncRNAs were found in RNA-seq data of multiple life stages of *Anopheles gambiae* [20]. These studies increased the catalog of insect lncRNAs and provided insight into their functions, such as cell differentiation, transcription regulation, and dosage compensation [1]. Compared with mRNA, lncRNA exhibits more tissue specific-expression in insects, indicating a specific function associated with these tissues [21].

LncRNAs can play crucial roles in many biological processes, such as cell differentiation and development [22, 23]. In *Drosophila*, lncRNAs were probable involved in molting because the mass of lncRNAs was significantly up-regulated in the late embryonic and larval stages [5]. Knockdown of lincRNA_1317 expression by RNA interference suppressed the replication of dengue virus in *Aedes aegypti*, demonstrating the essential role of the lncRNA in anti-viral defenses [24]. Genome location and co-expression analyses of protein-coding genes and lncRNAs revealed that several lncRNAs might be associated with fecundity and virulence in *Nilaparvata lugens* [1]. More interestingly, specific expression of lncRNAs among tissues suggested their associated functions. In *Locusta migratoria*, knockdown of a brain-specific lncRNA (*PAHAL*) by RNA interference reduced aggregation behavior [25]. Functional annotation of target genes of testis-specific lncRNAs from RNA-seq data indicated that they may participate in the spermatogenesis of *Bombyx mori* [26].

The melon fly, *Zeugodacus cucurbitae* (Coquillett), is one of the most destructive and troublesome agricultural pests [27, 28]. The genome of *Z. cucurbitae* has been sequenced and released [29], which provides sequence information for gene annotation and functional research. The genome-wide expression of genes during the developmental stages has also been analyzed by RNA-seq [30]. However, there is no information about lncRNAs or functional studies in *Z. cucurbitae*. In this study, 24 RNA-seq datasets were constructed from different tissues of female and male *Z. cucurbitae*, including midgut, Malpighian tubules, fat body, ovary, and testis. By the way, a total of 3124 lncRNAs were strictly identified from the RNA-seq data, and their features and characteristics were analyzed. Differentially expressed lncRNAs between tissues in female and male adults, as well as similar tissues in female and male adults, were analyzed. Tissue-specific lncRNAs were screened in female and male tissues based on their relative expression levels. GO and KEGG pathway enrichment analysis of targets of midgut-specific lncRNAs revealed unique functional annotations. Our findings create a catalog of lncRNAs in tissues of *Z. cucurbitae* and provide information that will be useful for further functional studies.

## Results

### Identification and characterization of lncRNAs

**Fig. 1:**
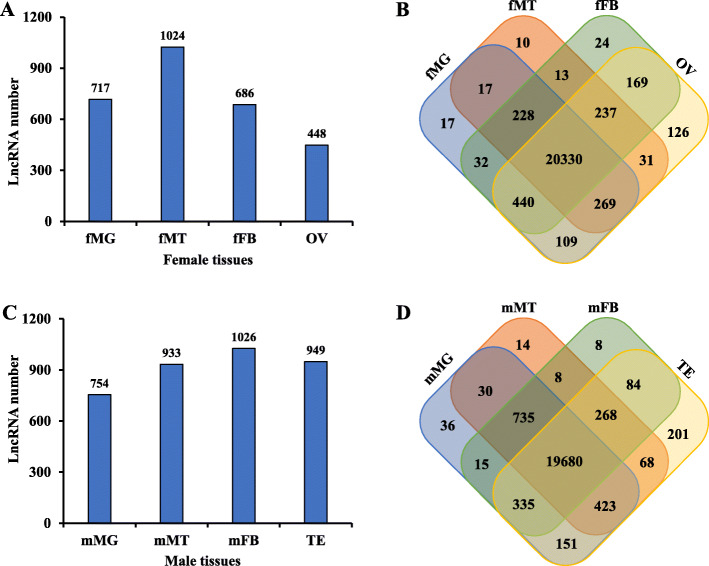
Number of lncRNAs in tissues of female (**a**) and male (**c**) *Zeugodacus cucurbitae*. LncRNAs with null expression in female tissues (**b**) and male tissues (**d**) were discarded. Abbreviations were consistent with the above

**Fig. 2:**
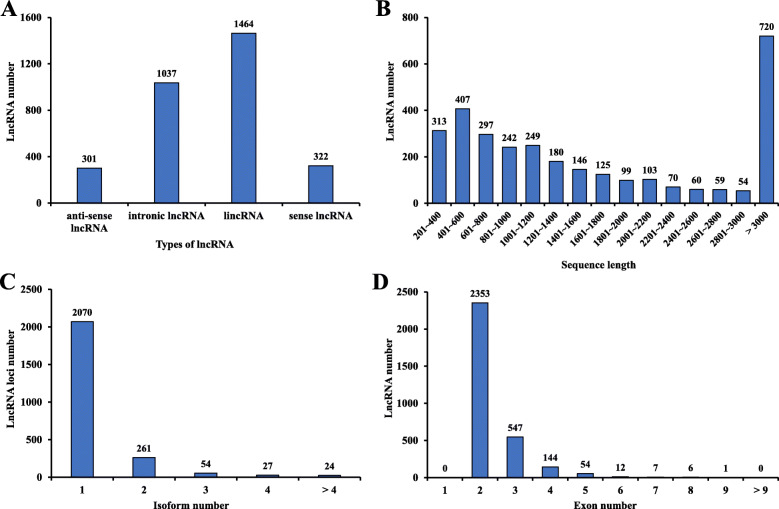
Number of four types of lncRNA (**a**), the lncRNA length distribution (**b**), the isoform number of lncRNA (**c**), the exon number distribution of lncRNA (**d**). lincRNA means long intergenic non-coding RNA

A total of 511,526,830 raw reads were generated from 24 RNA-seq datasets. Q30 scores were ≥ 93.0% in all of the samples. GC contents ranged from 40.1 to 46.69%. The accuracy of RNA-seq data was of high degrees as no “N” base was detected in any of the samples (Table 1). All of the RNA sequencing data produced in this study are available in the NCBI BioProject database (http://www.ncbi.nlm.nih.gov/bioproject/) under the accession number: PRJNA579200. After filtering under a computational pipeline (Fig. S1), a total of 22,159 lncRNA candidates were retained. Null-expressed transcripts (FPKM value < 1 in all analyzed samples) were discarded, and the numbers of lncRNAs in female and male tissues were screened. In females, the largest population of lncRNAs (1024) was found in the Malpighian tubules (Fig. 1a). There were 20,330 null-expressed lncRNAs in female tissues (Fig. 1b). Fat body had the largest lncRNA population (1026) among male tissues (Fig. 1c). Male tissues had 19,680 null-expressed lncRNAs (Fig. 1d). After discarding all null-expressed lncRNAs, a total of 3124 lncRNA transcripts were strictly identified from the transcriptome data of the eight tissues. Most of these were lincRNAs (1464; 46.9%), followed by intronic lncRNAs (1037; 33.2%), anti-sense lncRNAs (301; 9.6%), and sense lncRNA (322; 10.3%) (Fig. 2a). The lncRNA length distribution showed that most lncRNA transcripts were longer than 3000 nucleotides (Fig. 2b). The majority of lncRNAs only had one isoform (Fig. 2c). Most of the lncRNAs in *Z. cucurbitae* contained two exons (Fig. 2d).

**Table 1:** Summary statistics of the RNA-seq data

Sample ID	Read Sum	Base Sum	GC (%)	N (%)	Q30 (%)	Genome Mapping Rate
fFB1	71,005,430	21,094,233,520	43.55	0	93.12	82.34%
fFB2	71,920,846	21,287,840,586	43.52	0	93.54	82.50%
fFB3	69,864,623	20,811,064,364	43.52	0	93.23	81.43%
fMG1	65,842,842	19,680,348,232	43.11	0	93.39	33.85%
fMG2	58,622,426	17,525,507,334	40.38	0	93.40	21.06%
fMG3	91,469,944	27,328,946,668	43.28	0	93.78	20.44%
fMT1	76,741,248	22,856,925,352	42.55	0	93.57	76.87%
fMT2	83,141,124	24,796,064,764	42.18	0	93.22	71.47%
fMT3	66,939,800	19,978,132,492	41.35	0	93.19	35.27%
fOV1	61,518,157	18,365,855,324	42.82	0	93.78	84.97%
fOV2	54,295,717	16,193,760,614	42.71	0	93.21	87.65%
fOV3	54,641,144	16,335,419,334	43.13	0	93.09	84.49%
mFB1	76,375,481	22,756,855,810	43.17	0	93.50	83.55%
mFB2	63,341,864	18,878,214,080	42.64	0	93.28	80.40%
mFB3	62,003,794	18,453,417,274	42.44	0	93.52	73.90%
mMG1	90,288,388	26,975,723,848	45.28	0	93.85	25.70%
mMG2	68,545,617	20,457,951,046	46.69	0	93.58	38.44%
mMG3	64,956,243	19,317,213,164	46.69	0	94.01	32.30%
mMT1	60,513,113	18,076,644,312	42.84	0	93.54	80.45%
mMT2	72,498,489	21,656,770,372	42.47	0	93.60	78.64%
mMT3	68,670,761	20,523,605,726	40.10	0	93.37	39.75%
mTE1	57,330,203	17,072,620,942	42.36	0	93.31	84.01%
mTE2	64,218,906	19,189,876,708	42.48	0	93.53	87.38%
mTE3	60,939,729	18,190,973,854	42.43	0	93.61	86.87%

Note: Q30 refers to nucleotides with a quality value above 30 in reads. Genome mapping rate means the percentage of reads mapped to the reference genome

*Abbreviations: f/m* female/male, *MG* midgut, *MT* Malpighian tubules, *FB* fat body, *OV* ovary, *TE* testis

### Expression of lncRNAs in *Z*. *cucurbitae*

**Fig. 3:**
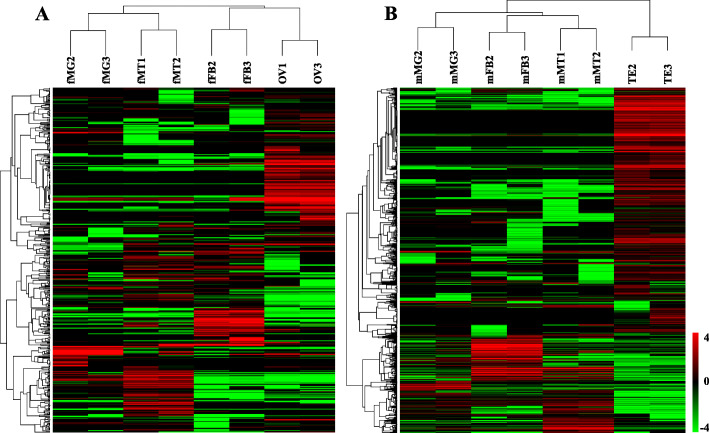
Cluster heatmap showing the expression profile of differentially expressed lncRNAs in female (**a**) and male (**b**) tissues of *Zeugodacus cucurbitae*. The heatmap was generated using R pheatmap. Red and Green indicate higher and lower expression levels, respectively. Abbreviations are consistent with the above

**Fig. 4:**
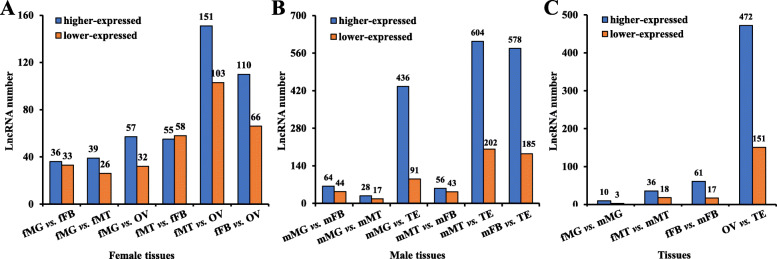
Statistical analysis of differentially expressed lncRNAs between tissues in female *Zeugodacus cucurbitae* (**a**), male *Zeugodacus cucurbitae* (**b**), and similar tissues between female and male *Zeugodacus cucurbitae* (**c**). Abbreviations are the same as above

To analyze the differences in expression of lncRNAs among tissues, the hierarchical clustering of 1554 differentially expressed lncRNAs (DELs) was analyzed in a heatmap using the FPKM value (Fig. 3). Many DELs clustered in specific tissues based on lncRNA expression levels among the different tissues. DELs between every two pairs of tissues were analyzed. In female *Z. cucurbitae*. A total of 151 higher- and 103 lower-expressed lncRNAs were found in the comparison of Malpighian tubules vs. ovary. The comparison of midgut vs. fat body showed 69 DELs, among which 36 were higher- and 33 were lower-expressed (Fig. 4a). Comparisons of Malpighian tubules vs. testis and midgut vs. Malpighian tubules had the most and fewest DELs in males, respectively. A total of 806 DELs were found in male Malpighian tubules vs. testis; 604 were higher- and 202 were lower-expressed. A total of 45 DELs existed in midgut vs. Malpighian tubules of males; 28 were higher- and 17 were lower-expressed (Fig. 4b). DELs between similar tissues in male and female adults were analyzed. The comparison of ovary vs. testis had 623 DELs, which was much more than other tissue comparisons (Fig. 4c).

**Fig. 5:**
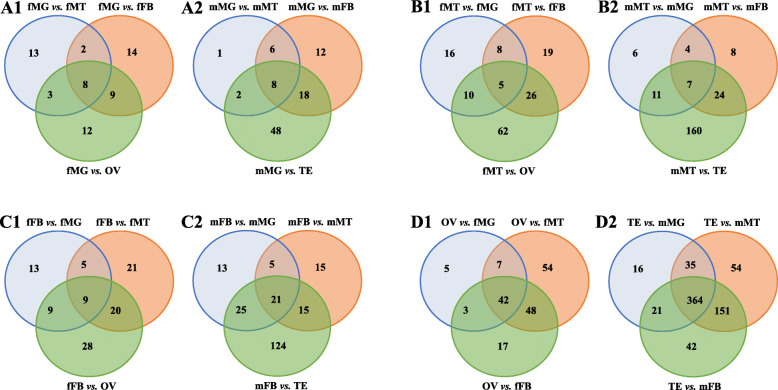
Quantitative expression analysis of midgut, Malpighian tubules, fat body, ovary, and testis in *Zeugodacus cucurbitae*. Each section of the Venn diagrams shows the numbers of differentially expressed lncRNAs with a ratio of two tissues expression level above 10. Venn diagrams indicate the number of midgut-specific lncRNAs (a1 and a2), Malpighian tubules-specific lncRNAs (b1 and b2), fat body-specific lncRNAs (c1 and c2), ovary-specific lncRNAs (d1), and testis-specific lncRNAs (d2) in female and male *Zeugodacus cucurbitae*. Abbreviations are consistent with those used previously

LncRNAs showed differential expression among tissues. Tissue-specific lncRNAs were identified in all tissues. Venn diagrams showed that each tissue contained a certain number of tissue-specific lncRNAs. In midgut, Venn diagram analysis showed 8 and 8 specifically expressed lncRNAs in females and males (Fig. 5a1 and a2). A total of 5, 7, 9, and 21 specifically expressed lncRNAs were found in female Malpighian tubules (Fig. 5b1), male Malpighian tubules (Fig. 5b2), female fat body (Fig. 5c1), and male fat body (Fig. 5c2), respectively. A total of 42 ovary-specific lncRNAs had a relatively high expression in the ovary compared with other female tissues (Fig. 5d1). The number of testis-specific lncRNAs (364) was much larger than those of other tissues (Fig. 5d2).

### Functional annotation of target genes of tissue-specific lncRNAs

**Fig. 6:**
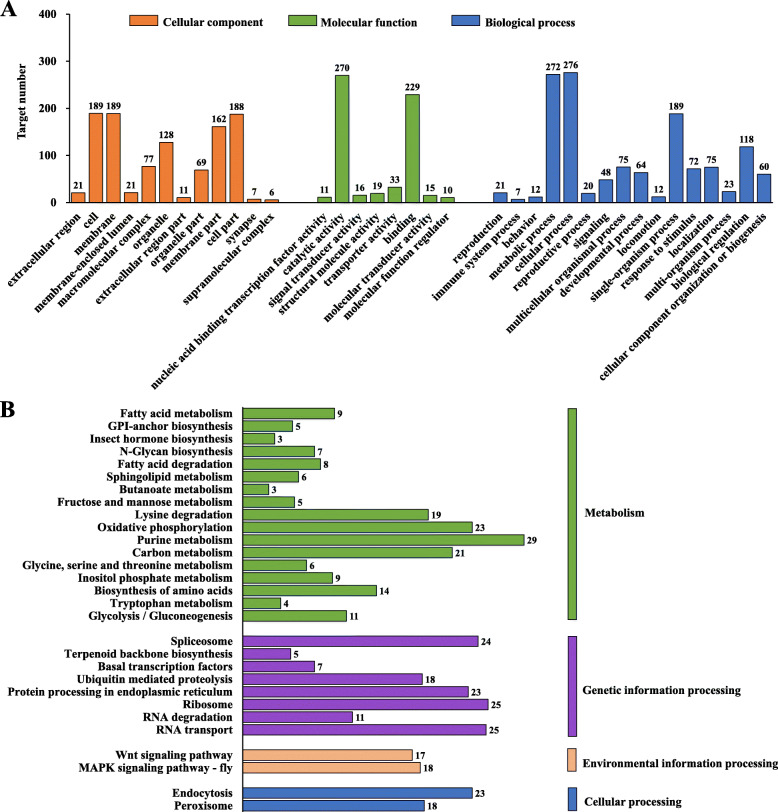
GO and KEGG pathway analyses of the target genes of midgut-specific lncRNAs in *Zeugodacus cucurbitae*. **a** GO analysis of the functions of lncRNA target genes. **b** KEGG pathway analysis of lncRNA target genes

GO and KEGG pathway analysis were conducted to study the potential functions of lncRNAs, and some of them can regulate the expression of neighboring genes (*cis*) and related co-expressed genes (*trans*) [31]. To illustrate some special functional annotations, target genes of tissue-specific (e.g., midgut-specific) lncRNAs were analyzed. A total of 457 target genes were obtained in the female midgut, among which 51 were *cis*-regulated and 410 were *trans*-regulated. For the male midgut, a total of 273 target genes were predicted, including 34 *cis*-regulated and 241 *trans*-regulated genes. GO analysis indicated that these target genes were involved in different physiological activities, including biological process, molecular function, and cellular component. In these categories, metabolic process, catalytic activity, and membrane were the most abundant subgroups (Fig. 6a). KEGG pathway analyses showed that these target genes were most frequently predicted in metabolism, among which the three pathways (purine metabolism, oxidative phosphorylation, and carbon metabolism) were most significantly enriched (Fig. 6b).

### Validation of differentially expressed lncRNAs

**Fig. 7:**
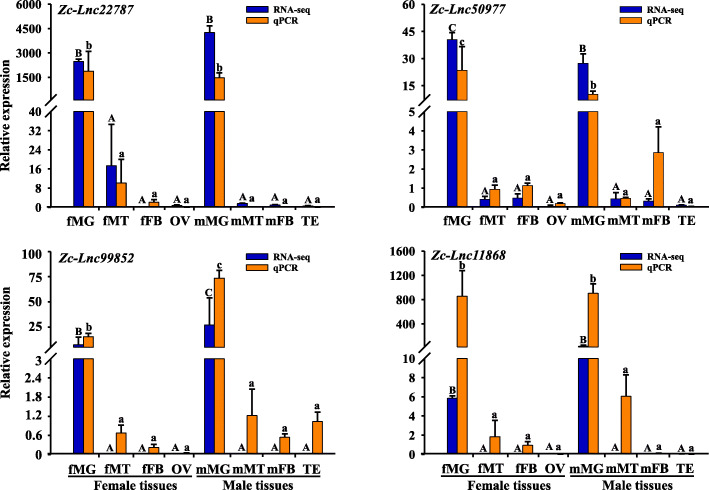
Validation of four randomly selected differentially expressed lncRNAs by quantitative real-time PCR (RT-qPCR). The bar represents the mean lncRNA expression and the error bar represents the positive standard error (SE) of the mean. Abbreviations were consistent with those used previously. Data were analyzed by one-way ANOVA followed with Tukey’s test (*P* < 0.05)

Four differentially expressed lncRNAs were randomly selected and their expression patterns in the eight tissues were examined by RT-qPCR. The selected four differentially expressed lncRNAs were named as *Zc-Lnc22787*, *Zc-Lnc50977*, *Zc-Lnc99852*, and *Zc-Lnc11868*. The expression patterns of these four lncRNAs calculated from RNA-seq data and RT-qPCR results were consistent (Fig. 7). All of our findings showed that our pipeline was strict in lncRNA identification and indicated that the identified lncRNAs were differentially expressed, in vivo*.*

## Discussion

The lncRNAs are responsible for several key physiological processes [32, 33], including epigenetics [34], immune response [35], and protein degradation [36]. LncRNAs in insect species have now been studied in *D. melanogaster* [5], *A. aegypti* [24], *B. mori* [26], *P. xylostella* [19], *N. lugens* [1], and *Phlebotomus perniciosus* [37]. The lncRNAs in *Z. cucurbitae* were undocumented, so we identified these lncRNAs and studied their expression in adult tissues by RNA-Seq.

Our transcriptome data were of high quality as illustrated by the relatively large Q30 percentages [30]. No “N” base was detected in any of the samples. It is possible that the presence of microbes in the *Z. cucurbitae* midgut resulted in the relatively low genome mapping rates. A low mapping rate was also reported in the midgut transcriptome of mosquito [38]. After identification under a computational pipeline, the screening criteria of the expression threshold of at least 1 FPKM in each tissue resulted in a strict catalog containing 3124 lncRNAs. A similar result was reported in *Drosophila*, in which 1077 lncRNAs were identified from 43,967 transcripts in the transcriptomes of different development stages [5]. Each tissue had a specific number of lncRNA in *Z. cucurbitae*. In *Drosophila*, lncRNAs were also distributed in many tissues of males [18]. Differences in the lncRNA numbers of different tissues may explain the variable lncRNA amounts in different insect species. Among the identified lncRNAs, the long intergenic lncRNAs (lincRNA) were most common, followed by intronic, sense, and anti-sense lncRNAs. In *B. mori*, lincRNAs and intronic lncRNAs were the most and least common, and sense lncRNAs were not identified [26]. Compared with *Z. cucurbitae*, a lack of sense lncRNA in *B. mori* was probably due to the different analytical methods used. In *Drosophila*, lincRNAs and sense lncRNAs were present in the largest and smallest numbers, respectively [39], which was consistent with our results. Blastn searches of *Z*. *cucurbitae* lncRNAs against NONCODE databases and NCBI nr were conducted, and no homologous sequences were detected. This demonstrated that lncRNA was not conserved among different species, which was consistent with previous studies [1, 26].

LncRNAs have shown similar molecular features and characteristics in different insect species. In the melon fly, the lengths of lncRNA transcripts were variable. The lncRNA group containing 201–600 nt had relatively more transcripts than other length intervals. The majority of lncRNA had two exons in *Z*. *cucurbitae*. Similarly, most abundant lncRNAs contained two exons in *N. lugens* [1]. The majority of *Drosophila* lncRNA transcripts contained 200–500 nucleotides [5]. The lncRNA group containing one isoform was the largest in *Z. cucurbitae*, which was consistent with the ENCODE project [40]. These results showed that *Z. cucurbitae* lncRNAs share features and characteristics similar to other insect lncRNAs.

LncRNAs showed various expression patterns in different insect tissues [21]. In female *Z. cucurbitae*, the comparison of Malpighian tubules vs. ovary owned the greatest amount of DELs, and this correlates with the large functional diversity between the two tissues. A similar result was reported in *B. mori* where the huge differential expression between posterior silk gland and testis correlated with a large functional difference [26]. DELs in comparisons containing testis were more abundant than in comparisons without testis in male *Z. cucurbitae* supporting the different expressions and functions of testis compared with other tissues. This is similar with *Drosophila*, in which testis owned the largest proportion of differentially expressed lncRNAs [41]. Additionally, DELs in ovary vs. testis were much more common than comparisons made between similar tissues. This reflects the large functional differences between the reproductive organs of female and male melon flies. Similarly, the ovary and testis showed the largest difference in *B. mori* [26]. As in *Drosophila* [39], the diverse distribution of lncRNAs in tissues of *Z. cucurbitae* resulted in extremely highly expressed lncRNAs in such tissues. Tissue-specific lncRNAs were obtained in female and male tissues of *Z. cucurbitae* based on their relative expressions. Each tissue contained specific lncRNAs indicating that their functions were associated with the target tissue [42]. *Drosophila* transcriptome analysis revealed that many lncRNAs had dominant expression in the testis [18], which was similar to our findings. Expression patterns of four randomly selected differentially expressed lncRNAs were determined by RT-qPCR, and the results of RNA-seq and RT-qPCR were consistent. This verifies the high quality of the RNA-seq [13].

The expression patterns of lncRNAs can help to clarify their possible biological roles. In *Drosophila*, knockout of 33 testis-specific lncRNAs by CRISPR/Cas9 reduced fertility [41]. A lncRNA (*CRG*), specifically expressed in the nervous system, regulates the locomotor ability and climbing ability of adult *Drosophila* [43]. In *Apis mellifera*, over-expression of an ovary-specific lncRNAs *lncov1* during a critical developmental period revealed its potential roles in regulating the ovary size of the worker bees [36]. In *B. mori*, functional annotation of Malpighian tubules-specific lncRNAs indicated integral components of membrane and oxidative phosphorylation were abundantly annotated, and fat body-specific lncRNAs suggested enrichment of the oxidation-reduction process and metabolic pathways [26]. Thus, the functions of lncRNAs were tightly associated with their specific distribution. In this study, functional annotations of targets of midgut-specific lncRNAs were analyzed. GO analyses revealed a frequent annotation of metabolic process, and KEGG pathway analyses showed that the majority of lncRNAs were annotated by metabolism pathways. Similarly, in *Anopheles gambiae*, metabolism was enriched in the functional annotation of lncRNA targets from the transcriptome of the midgut [44]. Thus, metabolism was enriched in midgut-specific lncRNA targets, indicating unique functions of the midgut and serving as a guideline for further functional research.

## Conclusion

We constructed 24 RNA-seq libraries from tissues of *Z. cucurbitae*, including midgut, Malpighian tubules, fat body, ovary, and testis. A total of 3124 lncRNA transcripts were qualitatively and quantitatively identified based on their expression. A total of 1554 differentially expressed lncRNAs were obtained, the greatest difference was found in Malpighian tubules vs. testis of males. Tissue-specific lncRNAs were identified in female and male *Z. cucurbitae* based on their relative expression levels. The most significant population of tissue-specific lncRNAs was found in testis. GO and KEGG pathway analysis revealed a special functional annotation of midgut-specific expressed lncRNA targets; metabolic process and metabolism were significantly enriched. This study released a informative catalog of lncRNAs in tissues of *Z*. *cucurbitae*, and the data will be useful for future functional studies.

## Methods

### Insects and tissue preparation

Melon flies were collected as pupae from Hainan Academy of Agricultural Sciences (20.01° N; 110.37° E), Haikou, Hainan Province, China, in 2016, and reared in an environmental chamber at 26 °C–27 °C and 65–75% relative humidity (RH) under a 14:10 h (light: dark) photoperiod in a temperature-controlled insectary [30]. Newly emerged melon fly adults were sexed and reared separately. Adults were dissected on day five to obtain the tissues, including midgut, Malpighian tubules, fat body, ovary, and testis. Each tissue was sampled separately from female and male adults with three biological replicates.

### RNA isolation, library construction, and sequencing

Total RNA was isolated from the 24 samples using TRIzol reagent (Invitrogen, Carlsbad, CA, USA) according to the manufacturer’s instructions. The concentrations of all RNA samples were tested with a NanoDrop One spectrophotometer (Thermo Fisher Scientific, Madison, WI, USA). The degrees of purity of RNA samples were measured by absorbance ratios of OD_260/280_ and OD_260/230_. The integrity levels were evaluated using 1% agarose gel electrophoresis.

A Ribo-Zero rRNA Removal Kit (Epicentre, Madison, WI, USA) was used to remove rRNA in the input material, which contained 1.5 μg RNA per sample. Sequencing libraries were constructed using the NEBNext® Ultra™ Directional RNA Library Prep Kit (NEB, Beverly, MA, USA) for Illumina sequencing following the manufacturer’s instructions. Index codes were added in order to attribute sequences to each sequencing sample. In this process, fragmentation was obtained using divalent cations under relative higher temperature using NEBNext First-Strand Synthesis Reaction Buffer (5×) (NEB). The random hexamer primers and reverse transcriptase (NEB) were used for first-strand cDNA synthesis. DNA Polymerase I and RNase H (NEB) were used for second-strand cDNA synthesis. The remaining overhangs were transformed into blunt ends via exonuclease/polymerase activities using exonuclease and polymerase (NEB). NEBNext Adaptor was ligated with a hairpin loop structure to prepare for hybridization after adenylation of the 3′-ends of the sequence fragments. Purification of library fragments were conducted with AMPure XP Beads (Beckman Coulter, Beverly, CA, USA), which generated fragments preferentially 150–200 bp in length. A total of 3 μL of USER Enzyme (NEB) was used in the procedure of size-selected and adaptor-ligated cDNA at 37 °C for 15 min. Then PCR was performed with Universal PCR primers, Phusion High-Fidelity DNA polymerase, and Index (X) Primer. In the end, an AMPure XP system (Beckman Coulter) was implemented in purification of PCR products, and evaluation of library quality was performed on an Agilent 2100 Bioanalyzer (Agilent, Palo Alto, CA, USA) [45]. After the libraries were prepared, sequencing was performed on an Illumina Hiseq platform by Biomarker Technologies (Beijing, China).

### Clustering, sequencing, and assembling

After cluster generation, sequencing of library and generation of paired-end reads were accomplished. The raw data produced from sequencing were firstly processed through in-house Perl scripts. The reads containing ploy-N or adapters and low-quality reads were removed so as to obtain clean reads. All of the downstream analysis were performed with clean data. Q20, Q30, GC percent, and the total number of clean data was computed in this step. The sequencing data were aligned to the *Z*. *cucurbitae* genome (ASM80634v1, GenBank assembly accession number: GCF_000806345.1) using TopHat program (version 2.0) [46], parameters “mismatch 2 (-N 2), Insert_size 40 (-r 40)” were used, and other parameters were default. Cufflinks (version 2.2.1) [47] and Scripture (versions VPaperR3) [48] software were used to assemble the final transcriptome, parameters “operation core number 4, library-type fr-unstrande” were used in Cufflinks and all the other parameters were default in the two softwares. Cuffdiff (version 2.1.1) was used to calculate the FPKM (fragments per kilobase per million reads) value of transcripts with default parameters [49], including lncRNAs and mRNAs in each sample.

### Identification of long non-coding RNAs

After the filtering and mapping, a step-wise filtering pipeline was developed to identify lncRNAs from the assembled transcriptome. In the first, transcripts shorter than 200 nt and those overlapped with protein-coding genes in the same sense of strand were discarded. At the same time, transcripts with open reading frames less than 300 nt and mapping to two more exons were retained. Next, three tools of Coding-Non-Coding Index (CNCI, version v2) [50], the Coding-Potential Assessment Tool (CPAT version 1.2.2) [51], and Coding Potential Calculator (CPC, version 0.9 r2) [52] were used to predict the protein-coding potential. Transcripts with CNCI scores < 0, CPAT = “no”, and CPC scores < 0 were retained. After that, Pfam was implemented and transcripts that contained any known protein domains would be excluded [53]. Finally, the remaining transcripts were aligned with Rfam database, GtRNAdb database, Silva database, and Repbase database, respectively, to screen out other ncRNA, such as small nuclear RNA (snRNA), transfer RNA (tRNA), small nucleolar RNA (snoRNA) repeat sequences, and ribosomal RNA (rRNA) using Bowtie tools [54]. Genome mapping rates revealed large differences among biological replicates of Malpighian tubules from female and male melon flies. Considering this, Malpighian tubules as well as other tissues had one deleted replicate, and the average FPKM values between the remaining two replicates were used for downstream analyses [20]. Transcripts with an FPKM value < 1 in all tissues were considered as null-expressed and were discarded. The remaining transcripts were considered reliable lncRNAs. Additionally, mRNAs were obtained from the same RNA-seq libraries in this study.

### Tissue-specific expressed lncRNAs

Tissue-specific lncRNAs refer to lncRNAs that have extremely high expression in the given tissue [18]. To study the tissue-specific lncRNAs in female and male *Z. cucurbitae*, DESeq was used to analyze the significance of the differential expression of lncRNAs in each two tissues [55]. In this step, the software provided a statistical program for calculating the difference in numeric gene expression analysis with fold change ≥2 and a False Discovery Rate (FDR) < 0.05. On this basis, tissue-specific expressed lncRNAs were screened in each tissue with the ratio of FPKM_tissue 1_/FPKM_all the others_ ≥ 10.

### Target prediction and GO and KEGG pathway analysis

LncRNA targets were predicted according to the genomic location and co-expression between lncRNAs and mRNAs. Two categories (*cis*-regulation and *trans*-regulation) of the lncRNA regulation modes were analyzed. LncRNAs’ regulation on their neighbor genes within 100 kb upstream and downstream in chromosomes was regarded as *cis*-regulation [56]. For *trans*-regulation, co-expression analyses of lncRNA and mRNA were investigated based on their expressions as previously implemented in tissues of *B. mori* [26]. Coefficients with *r* > 0.9 or < − 0.9 and pearson’s correlation with *p*-value < 0.01 were judged to be correlated expressed. All of the identified *cis*- and *trans*-regulated protein-coding genes were used for GO and KEGG pathway analysis. TopGO R packages and KOBAS software [57] were used for GO and KEGG pathway analysis, respectively.

### Quantitative real-time PCR (RT-qPCR)

To validate expression patterns of differentially expressed lncRNAs, the eight tissues were dissected from 5-day-old melon fly adults in the same manner as the sequenced samples. After total RNA isolation, lnRcute lncRNA cDNA kit (TIANGEN, Beijing, China) was used for first-strand lncRNA cDNA synthesis. Primers used for lncRNAs validation were designed using Primer 3.0 (http://bioinfo.ut.ee/primer3-0.4.0/) (Tab. S1). To determine the cycle threshold (*Ct*) value and amplification efficiency of each pair of primers, a standard curve was conducted with serial dilutions of cDNA (1, 5^− 1^, 5^− 2^, 5^− 3^, 5^− 4^). The qPCR reaction was run on a CFX384 Optics Module (Bio-Rad, Singapore) using the lnRcute lncRNA SYBR Green premix (TIANGEN, Beijing, China). RT-qPCR was conducted with 10 μL of mixture, each consisted of 5 μL of lncRNA SYBR premix, 4 μL of nuclease-free water, 0.5 μL of lncRNA cDNA (~ 500 ng/μL), and 0.25 μL each of forward and reverse primers (10 μM). The PCR procedure was as follows: an initial denaturation at 95 °C for 3 min, followed by 40 cycles of 95 °C for 5 s and 60 °C for 15 s, the specificity of primers were ensured by the record of a melting curve analysis from 60 °C to 95 °C. Relative expression levels of lncRNAs among different tissues were normalized by *Alpha-tubulin* and *beta-tubulin 1* [58]. All experiments were conducted in four biological replicates. Data were calculated by qBase plus software [59].

### Statistical analysis

The difference among tissues was analyzed using SPSS 19.0 software (IBM, Chicago, IL, USA) with a one-way analysis of variance (ANOVA) followed by Tukey’s honestly significant difference (HSD) test (*P* < 0.05).

## Supplementary information

**Additional file 1: Figure S1.** The computational pipeline for lncRNA identification from transcriptome.

**Additional file 2: Table S1.** Primer sequences used for RT-qPCR.

## Data Availability

All of the RNA sequencing data produced in the current study are available in the NCBI BioProject database (http://www.ncbi.nlm.nih.gov/bioproject/) under the accession number: PRJNA579200.

## Abbreviations

DEL: differentially expressed lncRNA
FB: fat body
FDR: false discovery rate
FPKM: fragments per kilobase per million reads
LincRNA: long intergenic non-coding RNA
LncRNA: long non-coding RNA
MG: midgut
MT: Malpighian tubules
nt: nucleotides
OV: ovary
TE: testis
vs.: versus
