# Supplementary figures and images for: Genome-wide analysis of long non-coding RNAs in adult tissues of the melon fly, Zeugodacus cucurbitae (Coquillett)
Source: BMC Genomics. 2020 Aug 31;21:600. doi: 10.1186/s12864-020-07014-x (PMC7457495; doi:10.1186/s12864-020-07014-x)

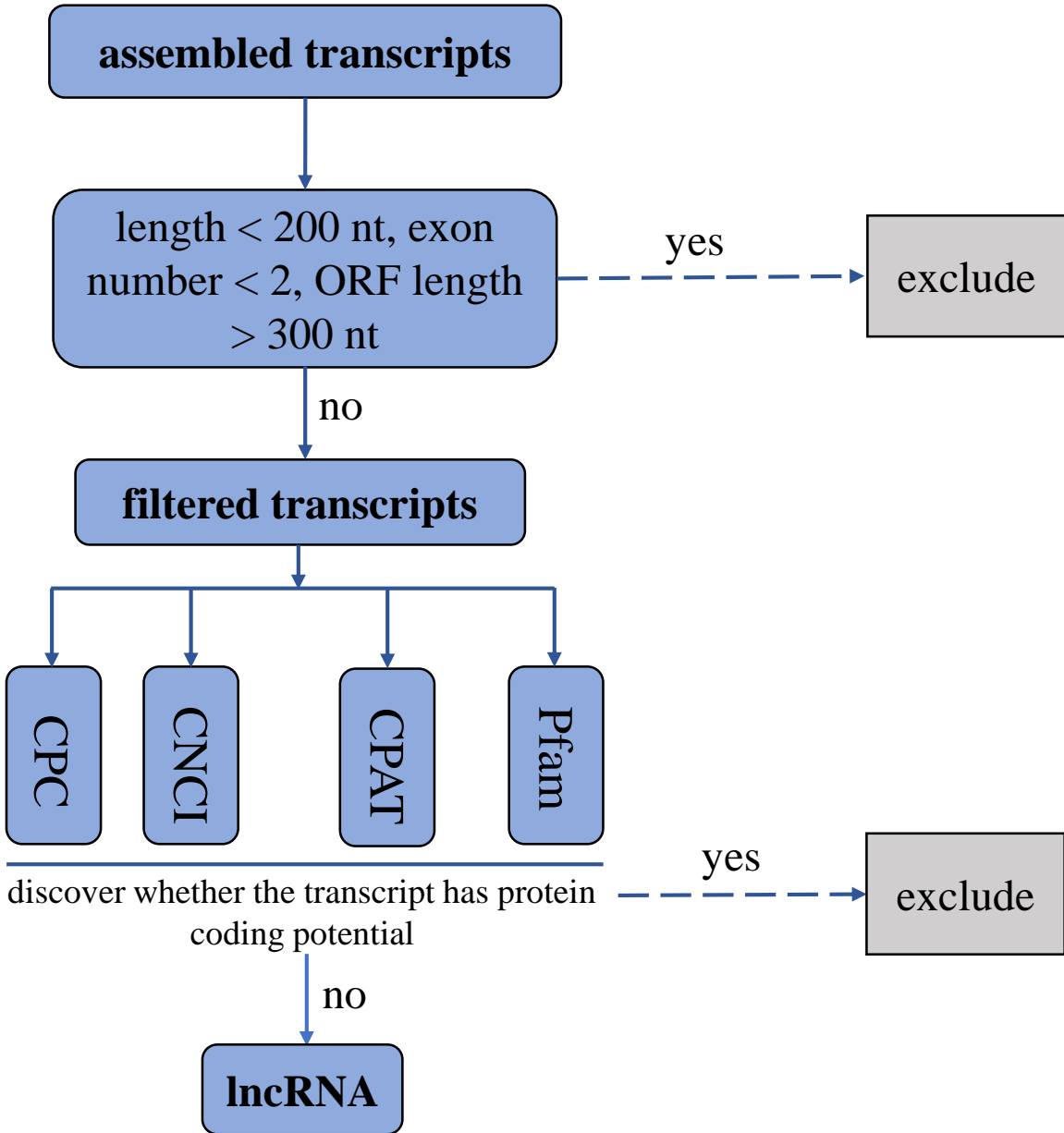

Supplement: Supplementary file 1 — Additional file 1: Figure S1. The computational pipeline for lncRNA identification from transcriptome. [file 12864_2020_7014_MOESM1_ESM.pdf]
